# Microbiome Profiling Using Shotgun Metagenomic Sequencing Identified Unique Microorganisms in COVID-19 Patients With Altered Gut Microbiota

**DOI:** 10.3389/fmicb.2021.712081

**Published:** 2021-10-11

**Authors:** Sijia Li, Siyuan Yang, Yuzheng Zhou, Cyrollah Disoma, Zijun Dong, Ashuai Du, Yongxing Zhang, Yong Chen, Weiliang Huang, Junru Chen, Deqiang Song, Zongpeng Chen, Pinjia Liu, Shiqin Li, Rong Zheng, Sixu Liu, Aroona Razzaq, Xuan Chen, Siyi Tao, Chengping Yu, Tianxu Feng, Wenyan Liao, Yousong Peng, Taijiao Jiang, Jufang Huang, Wei Wu, Liqiang Hu, Linghang Wang, Shanni Li, Zanxian Xia

**Affiliations:** ^1^Hunan Key Laboratory of Animal Models for Human Diseases, Department of Cell Biology, School of Life Sciences, Central South University, Changsha, China; ^2^Beijing Key Laboratory of Emerging Infectious Diseases, Beijing Ditan Hospital, Capital Medical University, Beijing, China; ^3^The First Hospital of Changsha, Changsha, China; ^4^Suzhou Geneworks Technology Co., Ltd., Suzhou, China; ^5^Xiangya School of Medicine, Central South University, Changsha, China; ^6^Department of Gynaecology and Obstetrics, The First Affiliated Hospital of University of South China, Hengyang, China; ^7^Hunan Provincial Key Laboratory of Medical Virology, Bioinformatics Center, College of Biology, Hunan University, Changsha, China; ^8^Center for Systems Medicine, Institute of Basic Medical Sciences, Chinese Academy of Medical Sciences and Peking Union Medical College, Beijing, China; ^9^Department of Plastic and Reconstructive Surgery, Shanghai Ninth People’s Hospital, Shanghai Jiao Tong University School of Medicine, Shanghai, China

**Keywords:** COVID-19, SARS-CoV-2, gut microbiota, gastrointestinal symptoms, microbiome

## Abstract

COVID-19 is mainly associated with respiratory distress syndrome, but a subset of patients often present gastrointestinal (GI) symptoms. Imbalances of gut microbiota have been previously linked to respiratory virus infection. Understanding how the gut–lung axis affects the progression of COVID-19 can provide a novel framework for therapies and management. In this study, we examined the gut microbiota of patients with COVID-19 (*n* = 47) and compared it to healthy controls (*n* = 19). Using shotgun metagenomic sequencing, we have identified four microorganisms unique in COVID-19 patients, namely *Streptococcus thermophilus*, *Bacteroides oleiciplenus*, *Fusobacterium ulcerans*, and *Prevotella bivia*. The abundances of *Bacteroides stercoris*, *B. vulgatus*, *B. massiliensis*, *Bifidobacterium longum*, *Streptococcus thermophilus*, *Lachnospiraceae bacterium* 5163FAA, *Prevotella bivia*, *Erysipelotrichaceae bacterium* 6145, and *Erysipelotrichaceae bacterium* 2244A were enriched in COVID-19 patients, whereas the abundances of *Clostridium nexile*, *Streptococcus salivarius*, *Coprococcus catus*, *Eubacterium hallii*, *Enterobacter aerogenes*, and *Adlercreutzia equolifaciens* were decreased (*p* < 0.05). The relative abundance of butyrate-producing *Roseburia inulinivorans* is evidently depleted in COVID-19 patients, while the relative abundances of *Paraprevotella* sp. and the probiotic *Streptococcus thermophilus* were increased. We further identified 30 KEGG orthology (KO) modules overrepresented, with 7 increasing and 23 decreasing modules. Notably, 15 optimal microbial markers were identified using the random forest model to have strong diagnostic potential in distinguishing COVID-19. Based on Spearman’s correlation, eight species were associated with eight clinical indices. Moreover, the increased abundance of *Bacteroidetes* and decreased abundance of *Firmicutes* were also found across clinical types of COVID-19. Our findings suggest that the alterations of gut microbiota in patients with COVID-19 may influence disease severity. Our COVID-19 classifier, which was cross-regionally verified, provides a proof of concept that a set of microbial species markers can distinguish the presence of COVID-19.

## Introduction

Coronavirus disease 19 (COVID-19) is caused by the highly infectious novel coronavirus severe acute respiratory syndrome coronavirus 2 (SARS-CoV-2), an enveloped and forward single-stranded RNA virus. SARS-CoV-2 is closely related to severe acute respiratory syndrome coronavirus (SARS-CoV) and Middle East respiratory syndrome coronavirus (MERS-CoV) (Jiang et al., 2020). After more than a year since the first case was identified, COVID-19 still remains a serious global public health crisis with a death toll surpassing 4 million globally (as of July 8, 2021). The main clinical features include fever, cough, and pneumonia, while some patients present diarrhea and other symptoms (Zhou F. et al., 2020). COVID-19 has emerged not only as a respiratory disease that mainly affects the lungs but also as a multiorgan disease that inflicts damage to other organ systems including the nervous and gastrointestinal (GI) systems (Mao et al., 2020; Patel et al., 2020; Tian J. et al., 2020; Wong et al., 2020).

A number of evidences have suggested that the GI system is important for the propensity and severity of COVID-19 (Jin et al., 2020; Lai et al., 2020; Villapol, 2020). Besides the typical respiratory and other physical symptoms, GI symptoms, e.g., nausea, vomiting, and diarrhea can also occur (Lerner et al., 2020; Tian Y. et al., 2020). In addition, SARS-CoV-2 has been detected in the tissues of the entire GI tract, and virus shedding in feces was documented in a significant proportion of patients even after negative RT-PCR in respiratory samples (Chen et al., 2020; Zhang et al., 2020). SARS-CoV-2 infection therefore has a direct impact on the GI system, possibly as an extrapulmonary site for virus replication and activity (Wolfel et al., 2020; Xu Y. et al., 2020). Infection in the GI system may impact the host immune responses. In recent studies, interleukin 18 (IL18), an important cytokine that mediates intestinal inflammatory reactions, is elevated in stool samples of patients (Tao et al., 2020). Also, up to 50% of fecal samples contain detectable viral RNA despite a negative result of rhinopharyngeal swab (Zuo et al., 2020a). Further emerging reports suggest that SARS-CoV-2 is able to infect the organoid model of the GI tract (Han et al., 2020; Krüger et al., 2020; Li, 2020).

Angiotensin-converting enzyme 2 (ACE2) serves as the critical receptor during viral entry of SARS-CoV-2 to the target host cells (Jiang et al., 2020; Letko et al., 2020). The spike protein of SARS-CoV-2 binds to ACE2 and is cleaved by the host serine protease TMPRSS2 (Hoffmann et al., 2020). In addition to respiratory epithelial cells, ACE2 and TMPRSS2 are also abundantly expressed in the ileum and colon (Zhang et al., 2020), especially in differentiated enterocytes (Burgueño et al., 2020; Ziegler et al., 2020). ACE2 is a homolog of ACE and has been described as a negative regulator of the renin–angiotensin system (RAS) (Verano-Braga et al., 2020). It mainly functions in pathological conditions related to excessive activation of RAS, including pathologies of the cardiovascular, renal, and pulmonary systems (Imai et al., 2006; Anguiano et al., 2017; Chatterjee et al., 2020). In human small intestinal organoids (hSIOs), SARS-CoV-2 and SARS-CoV can readily infect the enterocytes, which suggests that gut is also an important target organ of SARS-CoV-2 (Lamers et al., 2020). Diseases with impaired ACE2 expression or function may be contributing factors to intestinal dysbiosis (Perlot and Penninger, 2013). The intestinal dysbiosis is consistent with GI symptoms such as nausea and diarrhea reported in COVID-19 patients (Zhou F. et al., 2020), indicating the impact of SARS-CoV-2 infection on the GI system.

The integrity of the intestinal microbiome (the collective genome of the various microbiota of the human GI tract) may be disturbed by SARS-CoV-2, leading to intestinal malnutrition in the host. Intestinal malnutrition is also found in some other infectious diseases (Tuddenham and Sears, 2015). In the recent epidemiological reports, the highest morbidity and mortality due to COVID-19 are the elderly and those with underlying health conditions (Zhou F. et al., 2020; Zhou Z. et al., 2020). Interestingly, these populations tend to have a lower gut microbiota richness (Salazar et al., 2019; Yang et al., 2020). During hospitalization, COVID-19 patients had significant enrichment of opportunistic pathogens and depletion of beneficial commensals that persisted even after virus clearance and resolution of respiratory symptoms (Zuo et al., 2020b). When compared with H1N1 patients, the diversity and overall microbial composition are higher in COVID-19 patients (Gu et al., 2020). Previous studies, however, have various limitations including clinical type, small sample size, disease phase at the time of sample collection, and baseline microbial composition pre-infection. Therefore, it is important to expound on what main intestinal microbiota are associated with SARS-CoV-2 infection and how changes in the microbial composition impact the severity of COVID-19.

In this study, we prospectively collected 66 fecal samples from two cities in China and analyzed them by shotgun metagenomics sequencing. We investigated the diagnostic potential of gut microbiome for detection of COVID-19 using the random forest model. In the discovery cohort, we analyzed the gut microbiome characteristics of 10 healthy controls and 37 COVID-19 patients from Beijing and constructed a COVID-19 classifier. Then, we cross-regionally verified the diagnostic performance of the COVID-19 classifier in the validation cohort from Changsha, a central Chinese city about 1,400 km south of Beijing. In addition, we assessed the gut microbiome profiles of patients with different clinical types of COVID-19 and determined the correlation of certain microbial species with various clinical indices.

## Results

In this study, we prospectively collected 79 fecal samples from two regions in China (Table 1). After strict diagnosis and elimination procedures, 66 samples were selected for analysis, including 47 samples from Beijing (37 COVID-19 patients and 10 healthy controls) and 19 samples from Changsha (10 COVID-19 patients and 9 healthy controls). The samples from Beijing were used as the discovery cohort, while the samples from Changsha served as the validation cohort. In the discovery cohort, we characterized the gut microbiome of 37 patients and 10 healthy controls, identified key microbial markers, and constructed a COVID-19 classifiers using a random forest model. In the validation cohort, the diagnostic efficacy of the COVID-19 classifier was verified in 10 patients and 9 healthy controls. Our study only included COVID-19 cases confirmed by the nucleic acid test. The patient data were obtained from medical records and laboratory information systems including laboratory test results, clinical manifestations, and disease type (Supplementary Tables S1, S2). Moreover, the 47 samples of COVID-19 patients in the discovery and validation cohort were divided into four groups according to disease severity: mild, moderate, severe, and critical type (Xu Y.H. et al., 2020). The gut microbiome in these four groups was then characterized.

**TABLE 1:** The clinical characteristics of the participants in the discovery and validation cohort.

Clinical indices	Discovery cohort (*n* = 47)		*p*-value	Validation cohort (*n* = 19)		*p*-value
			
	Control (*n* = 10)	Disease (*n* = 37)		Control (*n* = 9)	Disease (*n* = 10)	
Age (years)	37.4 ± 9.43	44.05 ± 14.8	0.2071	46.78 ± 15.21	56.2 ± 14.49	0.2055
Sex (female/male)	3/7	18/19	0.3048	4/5	5/5	0.8504
Hospital stay (in days)	ND	35 ± 12.5	–	ND	35 ± 5.617	–
Sampling time (after hospitalization)	ND	8.35 ± 9.01	–	ND	31.4 ± 5.4	–
BMI	21.94 ± 2.341	23.17 ± 2.441	0.1528	23.19 ± 3.373	23.7 ± 4.133	0.9025
Known preexisting medical condition (yes/no)	0/10	4/33	–	0/9	6/4	–
Clinical type Mild Moderate Severe Critical	–	72253	–	–	0370	–
Stomach ache (yes/no)	–	3/34	–	–	1/8	–
Respiratory tract symptom (yes/no)	–	10/27	–	–	9/1	–
Antibiotic (yes/no)	–	12/25	–	–	6/4	–
Antiretroviral (yes/no)	–	0/37	–	–	10/0	–
TCM (yes/no)	–	26/11	–	–	1/9	–
Probiotics (yes/no)	–	0/37	–	–	5/5	–
WBC	5.602 ± 0.774	5.845 ± 1.890	0.7354	7.232 ± 0.922	4.539 ± 1.112	0.0007
Neutrophils	3.294 ± 0.601	3.548 ± 1.556	0.6541	4.12 ± 0.9044	2.981 ± 0.8671	0.0247
Lymphocytes	1.844 ± 0.270	1.781 ± 0.630	0.5074	2.41 ± 0.3187	1.174 ± 0.3735	0.0003
Monocyte	0.34 ± 0.074	0.4140 ± 0.1327	0.1487	0.4289 ± 0.08283	0.339 ± 0.1363	0.1629
Eosinophils	0.085 ± 0.034	0.08972 ± 0.1256	0.1961	0.2422 ± 0.1088	0.033 ± 0.05208	0.0007
Basophils	0.019 ± 0.018	0.01270 ± 0.01217	0.2649	0.03111 ± 0.01453	0.011 ± 0.007379	0.0025
RBC	4.886 + 0.507	4.758 ± 0.5292	0.4747	4.682 ± 0.4799	4.428 ± 0.634	0.4877
Hemoglobin	146.11 ± 17.42	144.0 ± 17.35	0.8250	146.1 ± 16.8	129.4 ± 19.26	0.0602
PLT	208.3 ± 49.24	219.3 ± 60.01	0.7354	227 ± 34.91	155.7 ± 53.93	0.0055
CREA	47.88 ± 13.16	65.98 ± 11.79	0.5556	67.18 ± 15	58.78 ± 12.94	0.1530
URCA	350 ± 92.68	337.5 ± 102.6	0.6558	338.7 ± 105	278 ± 71.1	0.2364
GLU	5.218 ± 1.370	5.385 ± 2.038	0.8518	5.016 ± 0.8887	6.843 ± 3.616	0.0662
ALT	26.96 ± 12.05	28.14 ± 19.55	0.5995	19.77 ± 11.43	14.69 ± 5.996	0.4379
AST	21.39 ± 6.220	26.71 ± 14.89	0.5242	22.66 ± 6.787	23.35 ± 9.088	0.9025
TBIL	11.14 ± 2.653	12.25 ± 5.241	0.6305	14.93 ± 3.676	13.21 ± 8.074	0.1530
DBIL	3.73 ± 1.161	4.808 ± 2.715	0.3765	4.644 ± 1.443	4.352 ± 3.02	0.3074
TP	72.25 ± 5.734	72.50 ± 5.447	0.8966	73.84 ± 2.235	66.75 ± 5.568	0.0142
ALB	43.03 ± 2.878	42.69 ± 3.638	0.5850	46.3 ± 1.681	39.1 ± 4.416	0.0013

*Continuous variables were expressed as mean ± standard deviation or median (interquartile range). Student’s *t*-test and Wilcoxon rank-sum test were used to compare continuous variables.*

*BMI, body mass index; TCM, traditional Chinese medicine; WBC, white blood cells; RBC, red blood cells; PLT, blood platelet; CREA, creatinine; URCA, uric acid; GLU, glucose; ALT, alanine aminotransferase; AST, aspartate aminotransferase; TBIL, total bilirubin; DBIL, direct bilirubin; TP, total protein; ALB, albumin; ND, no data; –, no calculation or no data.*

### Differences in Gut Microbial Diversity

**FIGURE 1:**
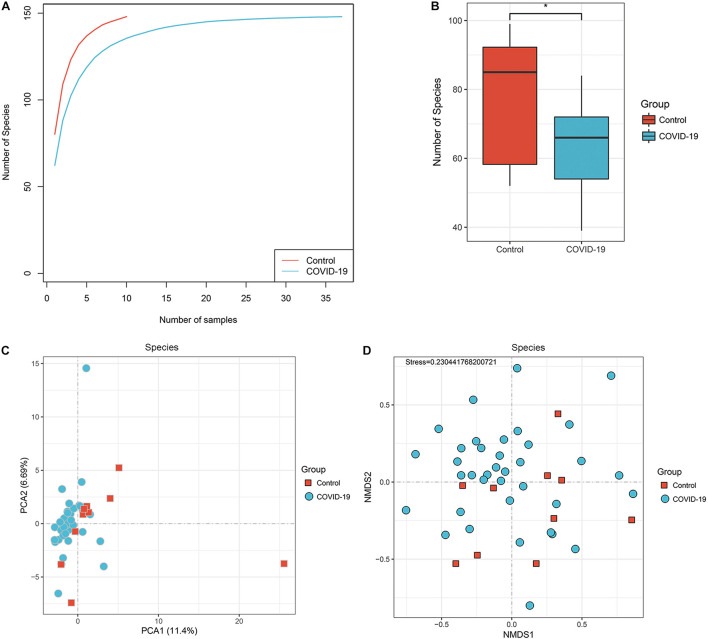
Differences in gut microbial diversity between COVID-19 and healthy controls (HC). **(A)** The rarefaction analysis between the number of samples and the number of species. As the number of samples increased, the number of species approached saturation in COVID-19 (*n* = 37) and HC (*n* = 10). **(B)** Compared with the HC, the number of species in COVID-19 was decreased significantly. *p < 0.05. **(C,D)** The principal component analysis (PCA) based on Bray–Curtis distance and the non-metric multidimensional scaling (NMDS) based on species level distribution showed that the gut taxonomic composition differed markedly between individuals in both COVID-19 patients and healthy controls (all *p* < 0.05; COVID-19).

To investigate the compositions and functions of the gut microbiome in patients with COVID-19, we sequenced the metagenome of 66 fecal samples (47 from patients with COVID-19 and 19 healthy controls). In the discovery cohort, a rarefaction analysis showed that the number of species richness nearly approached saturation as the number of samples increased in the COVID-19 group (*n* = 37). In the control group (*n* = 10), a similar but not exactly the same trend was observed, possibly due to a smaller sample size (Figure 1A). The number of species was significantly decreased in COVID-19 (*n* = 37) compared with healthy controls (*n* = 10; Figure 1B and Supplementary Figure S1).

In addition, the principal component analysis (PCA) is based on the distribution of the species level (Figure 1C, ANOSIM, *p* < 0.05) and the non-metric multidimensional scaling (NMDS) analysis (ANOSIM, Figure 1D) were conducted to illustrate the microbiome space of different samples. Considering the differences in gut microbiome composition among individuals, the samples of both groups were scattered rather than clustered (Figure 1D), but clustering is observed in PCA analysis (Figure 1C). The non-clustering is also consistent when principal co-ordinate analysis (PCoA), t-SNE, and UMAP were used (Supplementary Figure S1C–E). Additionally, we performed PCoA and NMDS separating antibiotic-treated and non-treated COVID-19 patients (Supplementary Figure S4).

### Altered Gut Microbial Compositions in Patients With COVID-19

**FIGURE 2:**
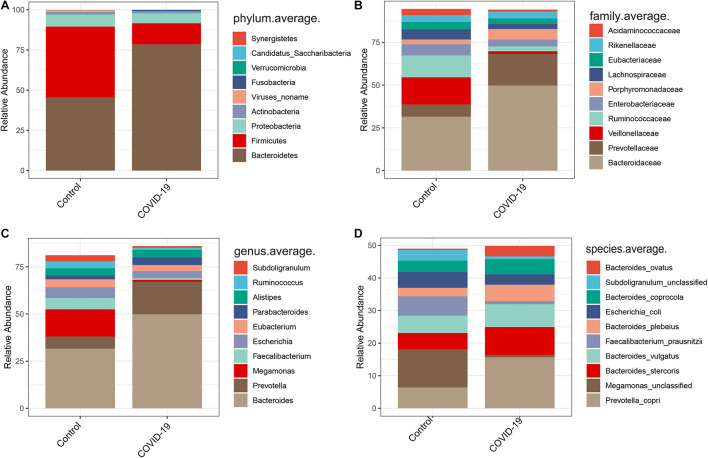
Altered gut microbial compositions in patients with COVID-19. **(A–D)** Average compositions and relative abundance of the bacterial community in COVID-19 and healthy control (HC) groups at the phylum, family, genus, and species levels.

In the discovery cohort, we further analyzed taxonomic compositions and alterations of the gut microbiome in participants from Beijing. Average compositions and relative abundance of the bacterial community in both groups at the phylum level showed that *Bacteroidetes*, *Firmicutes*, *Proteobacteria*, and *Actinobacteria* were dominant phyla in both COVID-19 patients and healthy controls (Figure 2A). Nonetheless, the phylum *Bacteroidetes* was evidently increased while the phylum *Firmicutes* was decreased in the COVID-19 group. Furthermore, *Candidatus saccharibacteria* was significantly reduced in COVID-19 patients versus healthy controls (*p* < 0.05, Supplementary Table S3).

**FIGURE 3:**
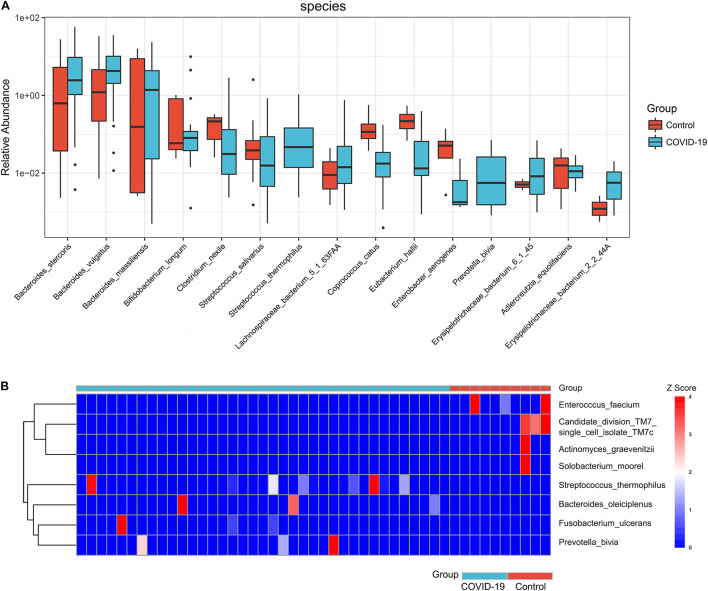
Significant species in patients with COVID-19. **(A)** Compared with HC (*n* = 10), eight species were significantly enriched, whereas seven species were significantly reduced in COVID-19 (*n* = 37) (all *p* < 0.05). **(B)** A heat map displaying the significantly abundant microbial species in COVID-19 (blue) and healthy control (red). Four species were unique to COVID-19 patients, whereas another four species were specific in HC (all *p* < 0.05).

Furthermore, we compared the gut microbial compositions between COVID-19 patients and healthy controls at the family, genus, and species levels. At the family level, *Candidatus saccharibacteria* and *Coriobacteriaceae* were significantly reduced in COVID-19 patients compared with those in the healthy controls (Figure 2B, all *p* < 0.05, Supplementary Table S4). Three genera including *Ruminococcus*, *Adlercreutzia*, and *Dorea* were significantly reduced in patients versus healthy controls (Figure 2C, all *p* < 0.05, Supplementary Table S5). At the species level, *Prevotella copri*, *Megamonas* sp., *Bacteroides stercoris*, *Bacteroides vulgatus*, *Faecalibacterium prausnitzii* (an anti-inflammatory bacterium), *Bacteroides plebeius*, *Escherichia coli*, *Bacteroides coprocola*, and *Subdoligranulum* sp. were dominant species in both COVID-19 patients and healthy controls (Figure 2D). Nine species including *Bacteroides stercoris*, *Bacteroides vulgatus*, *Bacteroides massiliensis*, *Bifidobacterium longum*, *Streptococcus thermophilus*, *Lachnospiraceae bacterium* 5163FAA, *Prevotella bivia*, *Erysipelotrichaceae bacterium* 6145, and *Erysipelotrichaceae bacterium* 2244A were significantly enriched, whereas six species, namely, *Clostridium nexile*, *Streptococcus salivarius*, *Coprococcus catus*, *Eubacterium hallii*, *Enterobacter aerogenes*, and *Adlercreutzia equolifaciens*, were significantly reduced in patients compared with those in the healthy controls (all *p* < 0.05, Figure 3A and Supplementary Table S6). Hence, our analyses revealed that patients with COVID-19 displayed obvious differences in their gut microbial composition. As shown in the heat map, four species including *Streptococcus thermophilus*, *Bacteroides oleiciplenus*, *Fusobacterium ulcerans*, and *Prevotella bivia* were unique to COVID-19 patients, whereas four other species including *Enterococcus faecium*, *Candidate division* TM7 single-cell isolate TM7c, *Actinomyces graevenitzii*, and *Solobacterium moorei* were specific in healthy controls (Figure 3B).

### Crucial KEGG Orthology and Pathways Related to COVID-19

**FIGURE 4:**
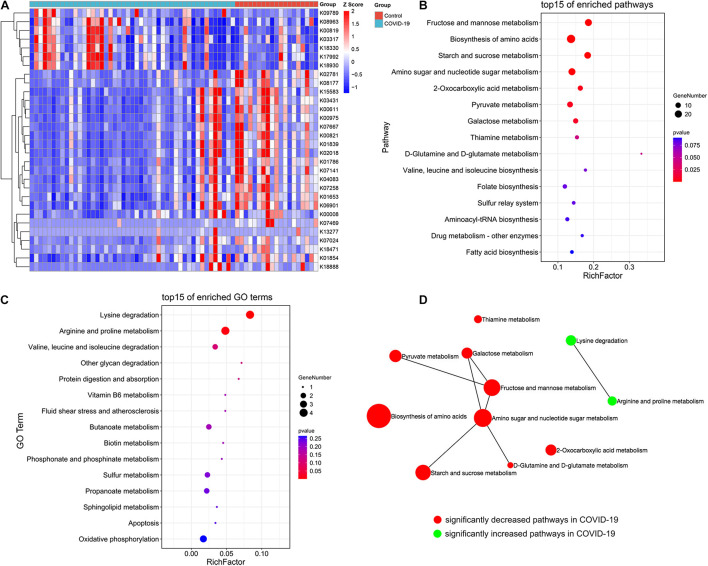
Crucial KEGG orthology and pathways related to COVID-19. **(A)** A heat map shows 30 KO modules overrepresented between the COVID-19 patients and healthy controls, including 7 increasing modules and 23 decreasing modules in disease patients. **(B)** A bubble chart showing the top 15 enriched pathways with 9 significantly decreased KEGG pathways in COVID-19. **(C)** A bubble chart showing the top 15 enriched GO terms with 2 significantly increased GO term in COVID-19. **(D)** A network of significantly increased and decreased pathways of COVID-19 patients.

The aforementioned results showed differences in microbiome composition between the COVID-19 patients and controls. To further investigate the functional roles of the altered intestinal microbiome in COVID-19 patients, the HUMAnN2 software was used to annotate the valid data of the samples, and the KO module abundance results and pathway abundance results were obtained. The functional enrichment analysis based on differential KO was used to explain the differences in metabolic pathways between the control group and the disease group through the hypergeometric distribution method. The gut microbial community function profiles and significantly different microbial functions in COVID-19 (*n* = 47) and the healthy controls (*n* = 19) are shown by a heat map (Figure 4A). Based on the MaAsLin analysis, 30 KO modules were overrepresented between the COVID-19 patients and healthy controls, including 7 increasing modules and 23 decreasing modules in disease patients. At the pathway level, nine metabolic pathways including D-glutamine and D-glutamate metabolism, fructose and mannose metabolism, starch and sucrose metabolism, 2-oxocarboxylic acid metabolism, thiamine metabolism, galactose metabolism, amino sugar and nucleotide sugar metabolism, biosynthesis of amino acids, and pyruvate metabolism were remarkably decreased, while two pathways including lysine degradation and arginine and proline metabolism were significantly increased in COVID-19 patients compared with those in the healthy controls (all *p* < 0.05, Figures 4B,C). We then constructed a network of significantly increased and decreased pathways in the COVID-19 patients (Figure 4D), which illustrated the connections between the enriched metabolic pathways.

### Diagnostic Potential of COVID-19 Based on the Gut Microbial Markers

**FIGURE 5:**
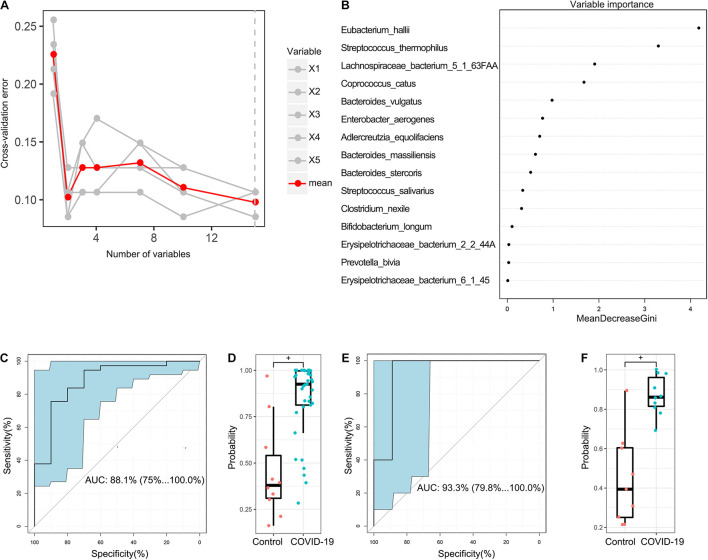
Diagnostic potential of gut microbial markers in COVID-19 patients. **(A)** A total of 15 microbial markers were selected as the optimal markers set by the random forest (RF) model. The cross-validation error curve was plotted based on 15 significant species. **(B)** Variable importance measured by mean decrease in accuracy of the 15 predictor species in the RF model. **(C)** ROC and AUC were calculated based on a test set of the discovery cohort. The AUC was 88.1% with 95% confidence interval (CI) of 75.1–100.0% between COVID-19 patients and healthy controls (*p* < 0.001). **(D)** Boxplot of probability in the discovery cohort. ^+^*P* < 0.01. **(E)** The AUC value in the validation cohort was 93.3% with a 95% CI of 75.1–100.0% between COVID-19 and healthy controls. **(F)** Boxplot of probability in validation cohort.

In the discovery cohort, a random forest classifier model between 37 COVID-19 patient samples and 10 healthy control samples was constructed to evaluate the potential of gut microbial markers as a non-invasive diagnostic tool for COVID-19. Through 10-fold cross-validation of the random forest model, 15 species were selected as the optimal marker set of COVID-19 (Figure 5A). Mean Decrease Accuracy was used to calculate the variable importance of 15 most important feature variables (Figure 5B). Receiver operating characteristic (ROC) curve analysis was used to calculate the confidence interval (CI) of ROC, and an area under the ROC curve (AUC) was determined by R. In the discovery cohort, the AUC value was 88.1% with 95% CI of 75.1–100.0% between COVID-19 patients and healthy controls (*p* < 0.001, Figure 5C). The box plot of probability indicated that the classifier model based on microbial markers reached a powerful diagnostic potential in distinguishing COVID-19 from healthy controls (*p* < 0.001, Figure 5D).

In addition, 9 healthy control samples were combined with 10 COVID-19 patient samples from Changsha to form a validation cohort. Using these combined samples of healthy and disease groups, we verified the diagnostic effectiveness of the classifier model for COVID-19. In the validation cohort, the AUC value was 93.3% with a 95% CI of 79.8–100.0% between COVID-19 and healthy controls (*p* < 0.001, Figure 5E). Moreover, the boxplot of probability also validated a significant diagnostic potential of gut microbial markers for COVID-19 (Figure 5F).

### Associations Between the Gut Microbiome and Clinical Indexes of COVID-19

**FIGURE 6:**
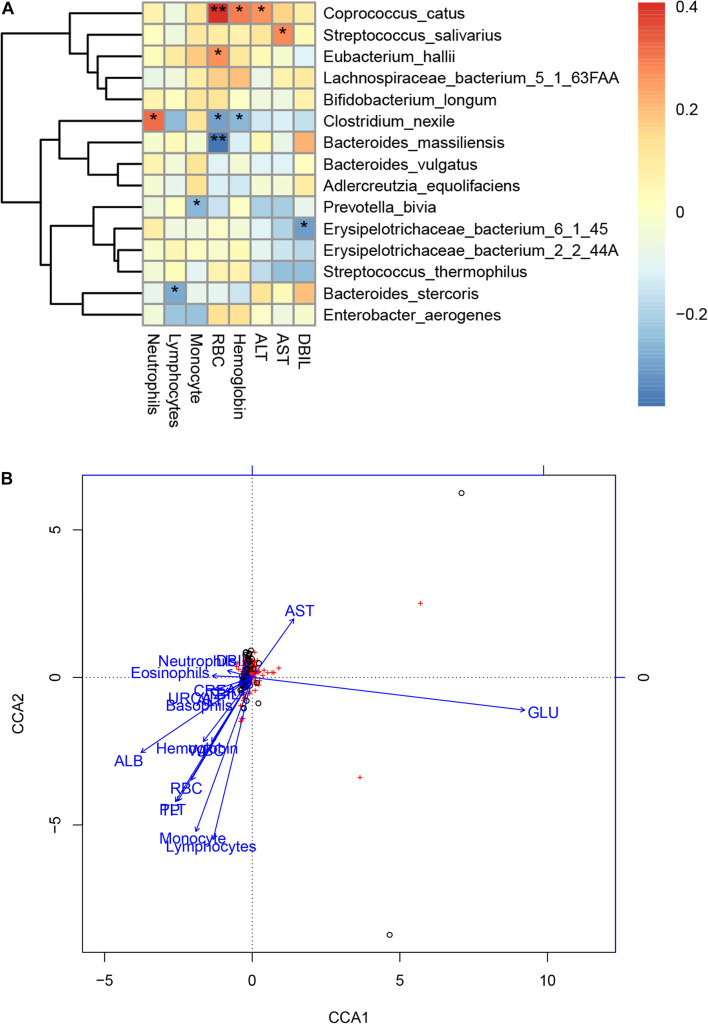
Associations between the gut microbiome and clinical indexes of COVID-19. **(A)** Canonical correlation analysis (CCA) between the gut microbiome and clinical indicators of COVID-19. The heat map shows the partial Spearman’s correlation coefficients of 15 microbial species markers with eight clinical indicators of COVID-19 (*n* = 47).*p < 0.05 and **p < 0.01. **(B)** A network of the associations between the gut microbiota and clinical indicators of COVID-19. RBC, red blood cells; DBIL, direct bilirubin; AST, aspartate aminotransferase; ALT, alanine aminotransferase; CREA, creatinine; URCA, uric acid; ND, no detection; –, no calculation or no data.

We further performed Spearman correlation analysis between the gut microbiota and clinical indexes of COVID-19 and found eight clinical indexes that are closely related to eight species of COVID-19 patients’ gut microbiome (Figure 6A). These are DBIL, hemoglobin, RBC, basophils, eosinophils, monocyte, lymphocytes, and neutrophils. Meanwhile, a network was made based on CCA to show the connections between gut microbiota and clinical indexes more clearly (Figure 6B). In addition, we also analyzed the CCA based on Pearson correlation analysis (Supplementary Figure S2). Among the associated indicators identified by Spearman correlation analysis, ALT, RBC, and hemoglobin level were positively correlated with *Coprococcus catus*. The AST level was positively correlated with *Streptococcus salivarius*. The RBC level was positively correlated with *Eubacterium hallii*. The neutrophil level was positively correlated with *Clostridium nexile*, while RBC and hemoglobin level were negatively correlated with it. The lymphocyte level was negatively correlated with *Bacteroides stercoris*. Moreover, the RBC level was negatively correlated with *Bacteroides massiliensis*. The monocyte level was negatively correlated with *Prevotella bivia*. DBIL was negatively correlated with *Erysipelotrichaceae bacterium* 6145 (Supplementary Table S7).

### Gut Microbiome Profiles Differ Across Clinical Types

**FIGURE 7:**
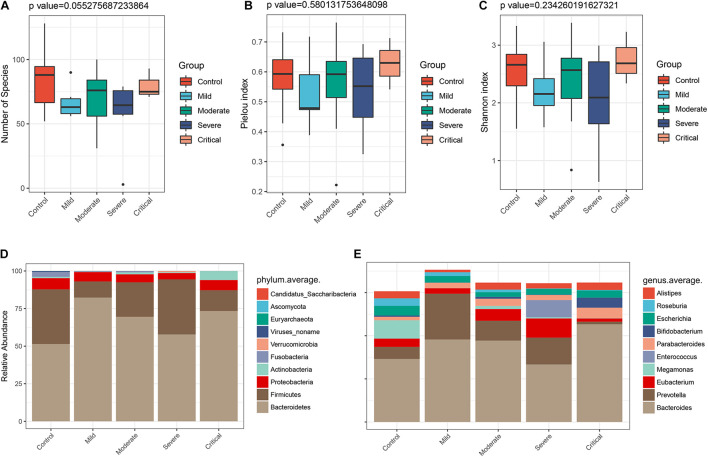
Gut microbiome profiles differ among various clinical types. The α diversity estimated by **(A)** number of species (*p* = 0.055), **(B)** Pielou index (*p* = 0.580), and **(C)** Shannon index (*p* = 0.234). **(D)** Average compositions and relative abundance of the bacterial community in four groups at the phylum level and **(E)** at the genus level.

To clarify the alterations of the gut microbiome in different clinical types, the 47 samples of COVID-19 patients in two cohorts were divided into four groups according to clinical types. They included seven samples of mild type (no radiographic evidence of pneumonia), 29 samples of moderate type (pneumonia was present), 8 samples of severe type (respiratory rate ≥ 30/min, or oxygen saturation ≤ 93% when breathing ambient air), and 3 samples of critical type (respiratory failure requiring mechanical ventilation, shock, or organ failure requiring intensive care). We characterized and compared the gut microbiome among these groups. As estimated by the alpha diversity index, the number of species (Figure 7A), Pielou index (Figure 7B), and the Shannon index (Figure 7C) were calculated. While there was no significant difference in gut microbial diversity among the four groups (Supplementary Figure S3), the disease groups collectively showed decreased number of species as compared to the healthy control. In terms of Pielou and Shannon indices, the mild type group seemed to be comparably lower than the healthy control. The average compositions and relative abundance of the bacterial community at the phylum level in all four groups are shown in Figure 7D, with *Bacteroidetes*, *Firmicutes*, *Proteobacteria*, and *Actinobacteria* as dominant phyla in all groups. The average compositions and relative abundance of the bacterial community at the genus level showed that *Bacteroidetes*, *Prevotella*, *Eubacterium*, *Megamonas*, *Enterococcus*, and *Parabacteroides* were dominant genera in four groups (Figure 7E).

**FIGURE 8:**
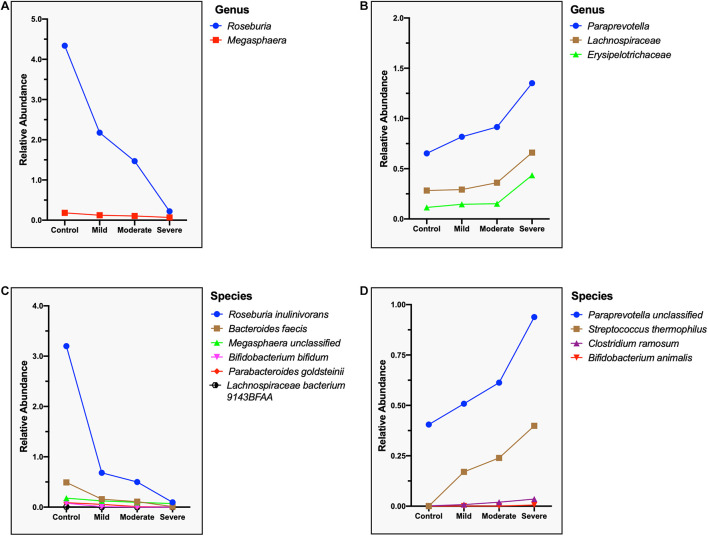
Microbial genera and species related to the severity of the COVID-19. **(A)** Negatively correlated microbial genus with the severity of COVID-19, showing a steep decrease in the relative abundance of *Roseburia*. **(B)** Positively correlated microbial genus with the severity of COVID-19, showing a sharp increase in the relative abundance of *Erysipelotrichaceae*. **(C)** Negatively correlated microbial species with the severity of COVID-19, showing a steep decrease in the relative abundance of *Roseburia inulinivorans*. **(D)** Positively correlated microbial species with the severity of COVID-19, showing a sharp increase in the relative abundance of *Paraprevotella* sp. and *Streptococcus thermophilus*.

Correspondingly, we calculated the relative abundance of three different clinical types of COVID-19 (excluding critical type, *n* = 3) and identified species that are positively and negatively correlated with the severity of COVID-19. Two genera, namely, *Roseburia* and *Megasphaer*, were negatively correlated with the severity of COVID-19 (Figure 8A), while *Paraprevotella*, *Lachnospiraceae*, and *Erysipelotrichaceae* were positively correlated with the severity of COVID-19 (Figure 8B and Supplementary Table S8). At the species level, six species including *Roseburia inulinivorans*, *Bacteroides faecis*, *Bifidobacterium bifidum*, *Parabacteroides goldsteinii*, *Lachnospiraceae bacterium* 9143BFAA, and *Megasphaera* sp. were negatively correlated with the severity of COVID-19 (Figure 8C). Because the relative abundance of butyrate-producing *Roseburia inulinivorans* is evidently depleted in COVID-19 patients, patients with COVID-19 may have a reconstruction of the butyrate metabolism pathway. Four species including *Paraprevotella* sp., *Streptococcus thermophilus*, *Clostridium ramosum*, and *Bifidobacterium animalis* were positively correlated with the severity of COVID-19 (Figure 8D and Supplementary Table S9).

## Discussion

The clinicopathology of COVID-19 has been previously described, but some aspects of the disease still need further research, e.g., the impact of altered gut microbiota (da Silva et al., 2020; Harrison et al., 2020; Huang et al., 2020). While there are reports about changes in microbial community during the disease presence (Zuo et al., 2020a,b; Chen et al., 2021; Yeoh et al., 2021), a consensus about the clinical relevance of these findings is still difficult to achieve. These studies have found that there are significant changes in the gut microbiota of COVID-19 patients, the α-diversity of the gut microbiota is decreased, and some conditional pathogens such as streptococcus have increased. Our work is a cross-regional study that comprehensively described the gut microbial profiling in four clinical types of COVID-19 patients. Based on the changes of microbial species, some important metabolic pathways were upregulated or downregulated in COVID-19 patients. Moreover, 15 most suitable COVID-19 microbial markers were identified through 10-fold cross-validation with a random forest model. The COVID-19 classifier based on microbial markers showed strong diagnostic potential in distinguishing COVID-19 patients from healthy controls. Our model has also successfully achieved a cross-regional verification. More notably, our correlation analyses determined nine species of gut microbiota related to 14 COVID-19 clinical indicators. In addition, we analyzed the gut microbiome of different clinical types of COVID-19 patients and identified the gut microbiota related to disease severity.

The abundance of two families and three genera in COVID-19 patients was significantly reduced. At the species level, nine species were enriched and six species were significantly reduced in COVID-19 patients. We have identified specific gut microbial species of COVID-19 patients and found a significant enrichment of opportunistic pathogens and pro-inflammatory bacterial species such as *Prevotella bivia* (Sharma et al., 2014), *Bacteroides* spp. (Wick and Sears, 2010), and *Erysipelotrichaceae* spp. (Kaakoush, 2015) in the intestines of COVID-19 patients. Our data suggest that probiotics and metabolism-promoting bacteria in the intestines of COVID-19 patients were significantly reduced, a finding that is consistent with previous reports (Jin et al., 2020; Zuo et al., 2020b). *Bacteroides stercoris*, *B. vulgatus*, and *B. massiliensis* were also identified to be enriched in COVID-19 patients. These *Bacteroides* spp. can downregulate the expression of ACE2 in the gut, resulting in enteric malnutrition (Perlot and Penninger, 2013; Zuo et al., 2020b).

Among the complications of COVID-19 is cytokine storm which is often observed in severely and critically ill patients. The high levels of cytokines, e.g., TNF, IL-6, and IL-1β, result in severe inflammation, causing damages to vital organs (Jose and Manuel, 2020). A recent report pointed out that the level of cytokine storm and inflammatory markers are associated with gut microbiota composition (Yeoh et al., 2021). This possibly contributes to the disease severity *via* modulation of host immune responses. However, further analysis is required to establish a direct causal relationship between the alterations of gut microbiome and hyperinflammation in COVID-19 patients. In pediatric patients, the autoimmune acute febrile inflammatory condition called Kawasaki’s disease is increasingly being reported as a complication associated with COVID-19 (Kabeerdoss et al., 2020; Ouldali et al., 2020). Kawasaki’s disease is characterized by a dysbiotic gut microbiome with increased levels of *Streptococcus* spp. (Esposito et al., 2019). In our study, the richness of proinflammatory bacterial species, including *Streptococcus thermophilus*, *Lachnospiraceae bacterium* 5163FAA, *Prevotella bivia*, *Erysipelotrichaceae bacterium* 6145, and *Erysipelotrichaceae bacterium* 2244A, were increased in COVID-19 patients. These changes therefore in the composition of gut microbiome may either initiate or enhance cytokine storm and other complications.

Furthermore, our results also showed that the composition of gut microbiome of four clinical types is different. We found that *Roseburia inulinivorans*, *Bacteroides faecis*, *Bifidobacterium bifidum*, *Parabacteroides goldsteinii*, *Lachnospiraceae bacterium 9143BFAA*, and *Megasphaera* sp. were negatively correlated with the severity of COVID-19, while *Paraprevotella* sp., *Streptococcus thermophilus*, *Clostridium ramosum*, and *Bifidobacterium animalis* were positively correlated. These data suggest that these microorganisms play an important role in the progression of COVID-19.

Recently, changes in metabolism have also been associated with COVID-19 following the increased prevalence of obesity and diabetes in severe cases (Drucker, 2021). It has been reported that in the sera of COVID-19 patients, amino acids and their derivatives were significantly decreased; meanwhile, over 100 lipids involved in lipid metabolism were downregulated in severe patients (Shen et al., 2020). In another study, fecal samples had a signature of an enhanced capacity for nucleotide and amino acid biosynthesis and carbohydrate metabolism (Esposito et al., 2019; Zuo et al., 2020a). Even in non-human primates, the alterations of gut microbiota during SARS-CoV-2 infection alter the bacteria’s metabolic output (Sokol et al., 2021). Patients from different areas or with different severities may have variant metabolic changes because of many factors, e.g., age, diet, and disease, which could affect metabolism. In our findings, the overall level of amino acid biosynthesis and some carbohydrate metabolism pathways were downregulated in COVID-19 patients. However, amino acid metabolism was upregulated specifically arginine and proline metabolism. Arginine is an amino acid involved in a number of biological processes, including the biosynthesis of proteins, host immune response, urea cycle, and nitric oxide production (Gambardella et al., 2020). This essential amino acid is mainly absorbed by the intestine (Tome, 2018). The increased arginine and proline metabolism might indicate intestinal dysfunction in COVID-19 patients. Considering that the metabolic changes in different patients are diverse or even opposite, more analyses are needed to understand the metabolic aspect of COVID-19 pathophysiology.

Lately, combination therapy has received interest. Dual or multiple therapies including microbial therapy have been seen to have a significant effect on certain diseases (Gutiérrez-Gutiérrez et al., 2017). A recent study has identified *Bifidobacterium pseudolongum* in the feces of mice as a marker bacterium for immunotherapy (Mager et al., 2020). Its metabolite inosine can promote Th1 cell differentiation and enhance the effect of immune checkpoint blockade (ICB) treatment in a specific environment. This provides proof of concept that immunotherapy combined with microbial therapy does have the ability to enhance the body’s anti-tumor immunity. In our work, *Bifidobacterium* sp. is significantly reduced in the COVID-19 patients’ fecal samples, possibly contributing to the decline of the patient’s immune response. Enhancing the level of this bacteria and thereby increasing its metabolite inosine may potentially improve the patient’s immune responses to SARS-CoV-2.

The main factors affecting the variation of the gut microbiome are geographical, diet, and population genetic factors (Zhernakova et al., 2016). This is why regional variation limits the application of intestinal microbial disease models to a certain extent (He et al., 2018). Our research, however, attempted to reduce the probable bias due to these factors. Our COVID-19 classifier model was cross-regionally verified. In part, our model reduces the influence of geographical, dietary, and genetic factors. Taken all our data together, we demonstrated the prospects of targeted biomarkers of gut microbiome as a non-invasive diagnostic tool for COVID-19. This new diagnostic tool can be used as a supplement to traditional diagnostic methods such as nucleic acid and serological tests.

Nonetheless, several limitations of this study should be discussed. Firstly, the small sample size of this study, especially in the healthy control group (*n* = 19), is a major limitation that may affect the findings. Secondly, the uneven distribution of patients according to disease severity also somehow limits our study to certain extent. Of the 47 patients with COVID-19, only three were critical cases. Further studies that include a larger population of critical cases are therefore recommended. Thirdly, although we have identified that some clinical markers were associated with specific microorganisms, such correlations require further analysis using transcriptomics and metabolomics to elucidate their clinical relevance. Fourthly, fecal samples were collected at only one time point during the whole course of hospitalization. Thus, it is unclear whether the altered gut microbiome of our patient cohort has persisted even after the resolution of symptoms. This is an interesting question that can be addressed in a future study. Fifthly, our study did not perform a correlation analysis between viral load and gut dysbiosis. In a previous report, patients with higher viral load also had higher abundance of opportunistic bacterial pathogens (Zuo et al., 2020a). However, such a correlation was not analyzed in our current work. Lastly, alteration of the gut microbiota may not be only caused by SARS-CoV-2 infection. Other respiratory syndrome, diseases, or coinfection of other pathogens could exert the same change in the gut microbial community alongside COVID-19 presence in a patient. Hence, further studies on this matter should be explored too.

The gut microbial composition of the disease and healthy groups of participants from Beijing and Changsha demonstrated the gut microbiome characteristics of COVID-19 patients. Specific microbial markers were identified, demonstrating their potential benefits as a non-invasive diagnostic tool for COVID-19. Meanwhile, the gut microbiota is altered across different clinical types of COVID-19 and distinct gut microbiota were associated with COVID-19 laboratory indicators. Our data provide a comprehensive survey of the gut microbiome in a relatively larger sample and provide evidence on the possibility of non-invasive biomarkers to diagnose COVID-19.

## Materials and Methods

### Study Cohort and Design

A total of 66 individuals were recruited in this study, including 47 patients with COVID-19 and 19 healthy controls matched for age, sex, and BMI. All individuals who received antibiotics, probiotics, or both within 4 weeks before enrollment were excluded. Diagnosis of COVID-19 infection was confirmed by real-time reverse transcription polymerase chain reaction (RT-PCR) of throat swab samples. SARS-CoV-2 RNA was detected using 2019-nCoV nucleic acid detection kit (Shanghai Berger Medical Technology Co., Ltd., China) following the manufacturer’s instruction. The demographic data (e.g., age, gender, and BMI), clinical manifestations, and treatment information of all patients and healthy controls were recorded. The study was approved by the Ethics Committee of Beijing Ditan Hospital and Changsha First Hospital (Number KT2020-045-03 and Number 1LX-2020049). Signed informed consent was obtained from all participants at the beginning of the study. All procedures were performed in accordance with the Declaration of Helsinki.

From January 2020 to March 2020, 10 patients with COVID-19 were admitted to Changsha First Hospital. From May 2020 to July 2020, 37 patients with COVID-19 were admitted to Beijing Ditan Hospital. The 7th edition of “Procedures for the Diagnosis and Treatment of COVID” was used to classify the disease severity into four categories: mild, moderate, severe, and critical. Mild cases presented mild clinical manifestations with no indications of pneumonia in imaging. Moderate cases are those with fever, respiratory symptoms, and pneumonia manifestations on imaging. Severe type manifested a respiratory rate (RR) of ≥30 beats/min, blood oxygen saturation ≤93% in resting state, partial pressure of oxygen (PaO2)/fraction of inspiration oxygen (FiO2) ≤ 300 mmHg, or presence of lesions in the lung imaging studies that has progressed significantly to >50% within 24–48 h. Critical cases manifested respiratory failure, requiring mechanical ventilation, shock, or other organ failure, requiring ICU monitoring and treatment. The healthy controls underwent routine complete blood count and other laboratory tests (Supplementary Tables S1, S2). The probiotics that were administered to some patients included *Bifidobacterium longum*, *Lactobacillus bulgaricus*, and *Streptococcus thermophilus*.

### Sample Collection and DNA Extraction

Fresh fecal samples were collected from the recruited subjects and were transported to hospital laboratory with an ice pack within 2 h. All samples were then frozen immediately and stored at −80°C prior to analyses.

DNA was extracted from the samples using the QIAamp DNA Stool Mini Kit (Qiagen, Hilden, Germany) according to the manufacturer’s instructions. Briefly, 20 μl proteinase K solution (20 mg/ml) and 100 mg zirconium beads (0.1 mm) were added to the pellet before the mixture was fully homogenized on a Mini-Beadbeater (FastPrep, Thermo Electron Corporation, Waltham, MA, United States) and then supplemented with buffer AL. The resulting mixture was incubated at 70°C for 10 min and supplemented with 200 μl ethanol (96%) before being loaded onto the QIAamp Mini spin column and centrifuged at 8,000 *g* for 1 min. The column was washed successively with 500 μl buffer AW1 and 500 μl buffer AW2. Finally, DNA was eluted with 100 μl buffer AE. DNA was quantified with a Qubit Fluorometer by using Qubit dsDNA BR Assay kit (Invitrogen, United States), and the quality was checked by running aliquot on 1% agarose gel.

### Library Construction and Sequencing

One microgram of genomic DNA was randomly fragmented by Covaris. Magnetic beads were used to select fragmented genomes with an average size of 300–400 bp. DNA was quantified by a Qubit fluorometer. The fragments were subjected to end-repair and then were 3′ adenylated. Adaptors were ligated to the ends of these 3′ adenylated fragments. The double-stranded products were heat denatured and circularized by the splint oligo sequence. The single-strand circle DNA (ssCir DNA) was formatted as the final library. The library was then qualified by the Agilent Technologies 2100 Bioanalyzer.

The qualified libraries were sequenced on a BGISEQ-500 platform (BGI Shenzhen, China). The library was amplified to make DNA nanoballs (DNBs) with more than 300 copies of one molecule. The DNBs were loaded into the patterned nanoarray, and paired-end 150 base reads were generated in the way of sequenced by combinatorial probe-anchor synthesis (cPAS).

### Quality Control and Metagenomic Profiling

After quality control of raw sequencing reads by using SOAPnuke (Chen et al., 2017) (v1.5.6, parameters: −l 15, −q 0.2, −n 0.05 −T 8), reads aligned to the human genome [alignment with SOAP2 (Li et al., 2009), Version 2.21, parameters: −p 6 −r 2 −m 200 −x 400] were removed. Then, the remaining high-quality reads were used for taxonomic and functional profiling utilizing MetaPhlan2 (Truong et al., 2016) and HUMANn2 (Franzosa et al., 2018) with default settings. An average of 38,464,387 reads per sample was obtained (minimum: 15,269,814; maximum, 51,836,736; Supplementary Table S10).

### Statistical Analysis

Continuous variables were expressed as mean ± standard deviation. The Student’s *t*-test and Wilcoxon rank-sum test were used to comparing continuous variables. To evaluate species richness of samples, within-sample (α) diversity indices including Chao1 index, number of species, Pielou index, and Shannon index were calculated based on the species profile using vegan package in R software (Version 3.3.3). Random sampling 100 times in the cohort with replacement from a given number of samples was performed, and the total number of species was identified from these samples by R (Version 3.3.3, vegan package). In order to study the differences in sample microbial communities between groups, NMDS and anosim were analyzed using the vegan package in R. PCoA was performed by ape package and vegan package in R.

Significantly different phyla, genera, species, and KEGG orthologs were identified using MaAsLin (Morgan et al., 2012). The alpha diversity index was tested by the Wilcoxon rank-sum test, and p values were corrected by the FDR method. Only features that existed in at least 10% of subjects were considered in the analyses. Based on the clinical indices and different species profiles, Spearman’s correlation was calculated in all samples. The *p*-values were corrected by R (Version 3.3.3 psych package). Functional enrichment analysis based on differential KOs was executed to illustrate the difference in metabolic pathways between the control group and the disease group by the hypergeometric distribution method.

Based on 15 differential species, a random forest classifier was trained on data of the discovery cohort using the random forest package in R. Ten-fold cross-validation was used to evaluate the performance of the predictive model. In the cross-validation error curve, the number of variables was 15 at the lowest cross-validation error. Finally, the 15 most important variables were selected to construct the prediction model. ROC curve analysis is used to calculate AUC and the confidence interval of ROC by R (Version 3.3.3 pROC package), and the validation cohort is used to measure the performance of the model (Li et al., 2017).

To determine the relative abundance of the various taxa at genus and species levels, the abundances of these taxa from each sample in the same group (control, mild, moderate, and severe) were pooled. Thus no error tick to variability is a limitation in Figure 8.

## Data Availability Statement

Shotgun microbial metagenome sequences examined in this work were deposited in the European Nucleotide Archive (https://www.ebi.ac.uk/ena/browser/home) with accession ID PRJEB43555. The dataset will be publicly available upon publication of this manuscript. All other data are available from the authors upon reasonable request.

## Ethics Statement

The studies involving human participants were reviewed and approved by Beijing Ditan Hospital, Capital Medical University. The patients/participants provided their written informed consent to participate in this study. Written informed consent was obtained from the individual(s) for the publication of any potentially identifiable images or data included in this manuscript.

## Author Contributions

SLi collected and classified the samples, organized patients’ information, and wrote the manuscript. SY, YuZ, and CD analyzed the data and wrote the manuscript. ZD, ZC, PL, SLiu, XC, TF, ST, CY, AR, WL, and YoZ searched for relevant literature. YC, WH, SY, RZ, AD, ShiL, and LH collected patients’ information and processed patients’ samples. JC and DS did the bioinformatic analysis. TJ, LW, LH, ShaL, WW, JH, YP, and ZX conceived the study and designed the project. All the authors read and approved the final manuscript. ZX led the project.

## Conflict of Interest

JC and DS are employed by Suzhou Geneworks Technology Co., Ltd. The remaining authors declare that the research was conducted in the absence of any commercial or financial relationships that could be construed as a potential conflict of interest.

## Publisher’s Note

All claims expressed in this article are solely those of the authors and do not necessarily represent those of their affiliated organizations, or those of the publisher, the editors and the reviewers. Any product that may be evaluated in this article, or claim that may be made by its manufacturer, is not guaranteed or endorsed by the publisher.

## Supplementary Material

Supplementary Figure S1 Gut microbiome profiles of the discovery cohort (*n* = 37). The α diversity estimated by **(A)** Shannon index and **(B)** Pielou index. The β diversity estimated by **(C)** PCoA, **(D)** T-SNE, and **(E)** UMAP.

Supplementary Figure S2 Canonical correlation analysis (CCA) between the gut microbiota and clinical indexes by Pearson correlation analysis of all COVID-19 patients (*n* = 47).

Supplementary Figure S3 The β diversity of various clinical types.

Supplementary Figure S4 The β diversity of ABX-treated and non-treated patients.

Supplementary Table S1 The laboratory results of complete blood count and blood chemistry of COVID-19 patients (*n* = 47) and healthy controls (*n* = 19).

Supplementary Table S2 Demographic profiles and drug treatment history of study participants.

Supplementary Table S3 Gut microbial compositions at phylum level of the discovery cohort (*n* = 37).

Supplementary Table S4 Gut microbial compositions at family level of the discovery cohort (n = 37).

Supplementary Table S5 Gut microbial compositions at genus level of the discovery cohort (*n* = 37).

Supplementary Table S6 Gut microbial compositions at species level of the discovery cohort (*n* = 37).

Supplementary Table S7 Correlation analysis of 15 gut microbial markers with various clinical indices of COVID-19.

Supplementary Table S8 Relative abundance of gut microbiota at the genus level in the different clinical types of COVID-19.

Supplementary Table S9 Relative abundance of gut microbiota at the species level in the different clinical types of COVID-19.

Supplementary Table S10 Sequencing information of 66 samples.

## Abbreviations

SARS-CoV-2: severe acute respiratory syndrome coronavirus 2
COVID-19: coronavirus disease 2019
SARS-CoV: severe acute respiratory syndrome coronavirus
MERS-CoV: Middle East respiratory syndrome coronavirus
GI: gastrointestinal
ACE2: angiotensin-converting enzyme 2
RAS: renin–angiotensin system
RR: respiratory rate
PaO2: partial pressure of oxygen
FiO2: fraction of inspiration oxygen
DNB: DNA nanoball
cPAS: combinatorial probe-anchor synthesis
BMI: body mass index
KO: KEGG orthology
ROC: receiver operating characteristic curve
CI: confidence interval
AUC: an area under the receiver operating characteristic curve
CCA: canonical correlation analysis.
