# Supplementary figures and images for: Microbiome Profiling Using Shotgun Metagenomic Sequencing Identified Unique Microorganisms in COVID-19 Patients With Altered Gut Microbiota
Source: Front Microbiol. 2021 Oct 11;12:712081. doi: 10.3389/fmicb.2021.712081 (PMC8542975; doi:10.3389/fmicb.2021.712081)

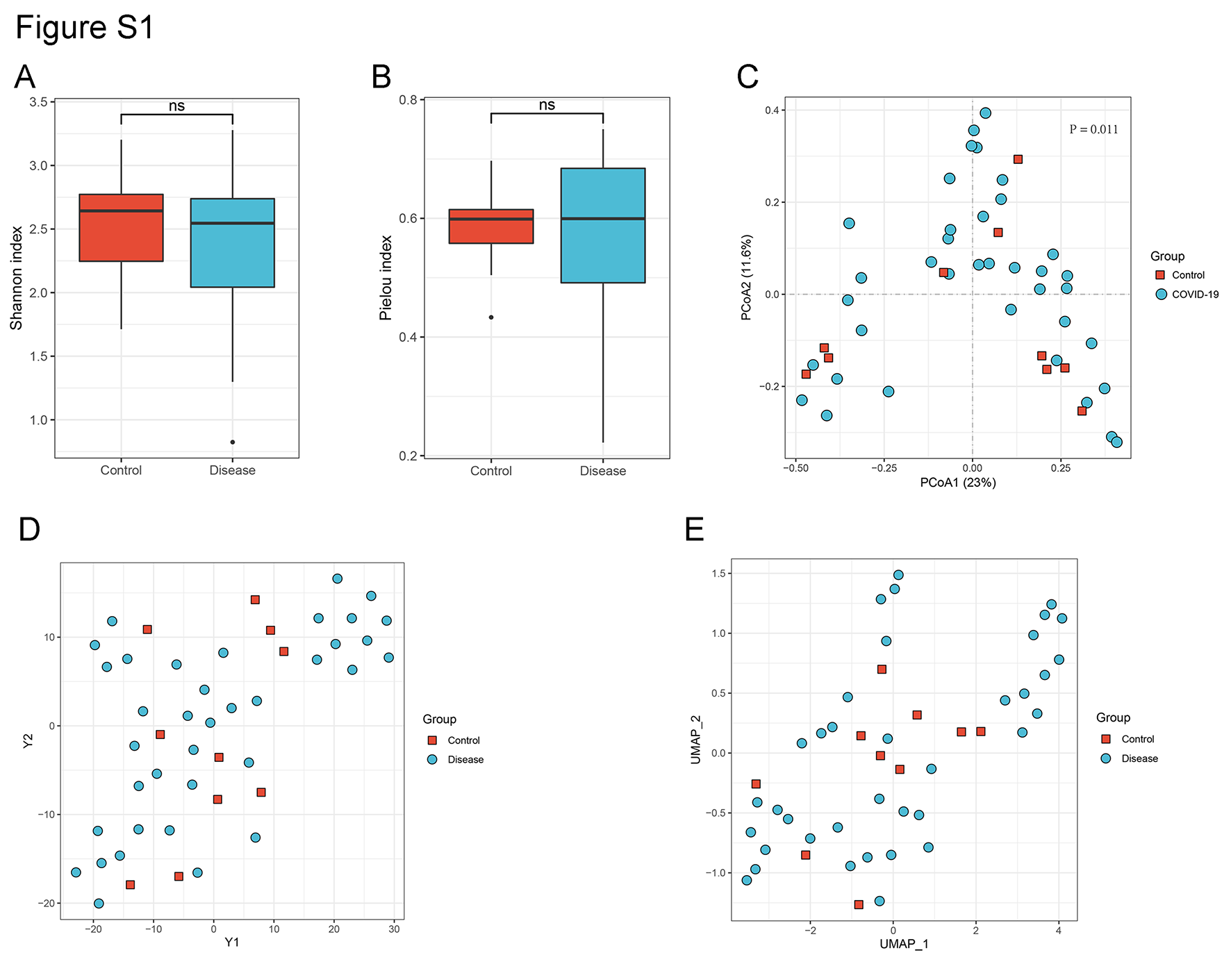

Supplement: Supplementary Figure S1 — Gut microbiome profiles of the discovery cohort (n = 37). The α diversity estimated by (A) Shannon index and (B) Pielou index. The β diversity estimated by (C) PCoA, (D) T-SNE, and (E) UMAP. [file Image_1.TIF]

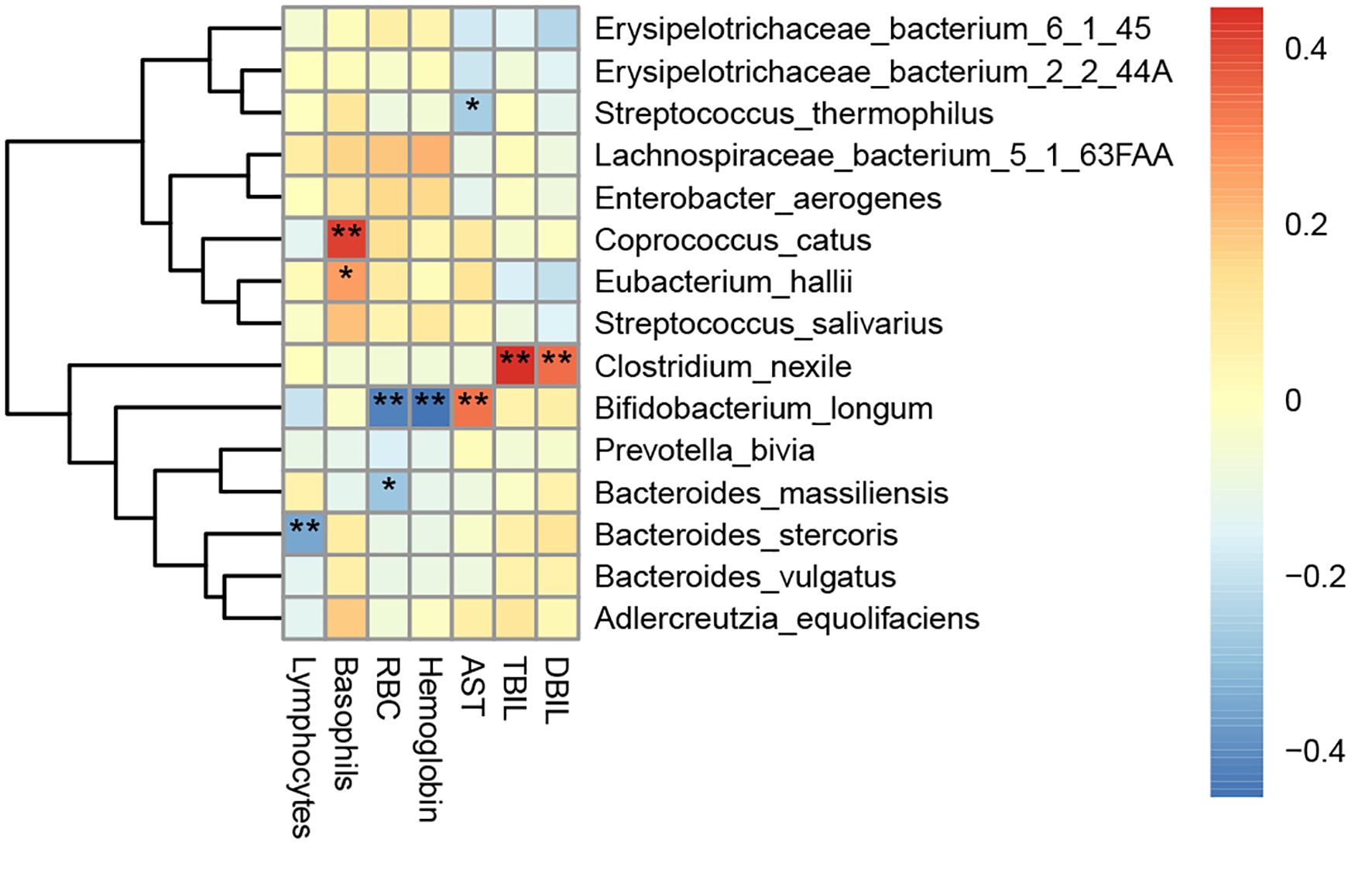

Supplement: Supplementary Figure S2 — Canonical correlation analysis (CCA) between the gut microbiota and clinical indexes by Pearson correlation analysis of all COVID-19 patients (n = 47). [file Image_2.TIF]

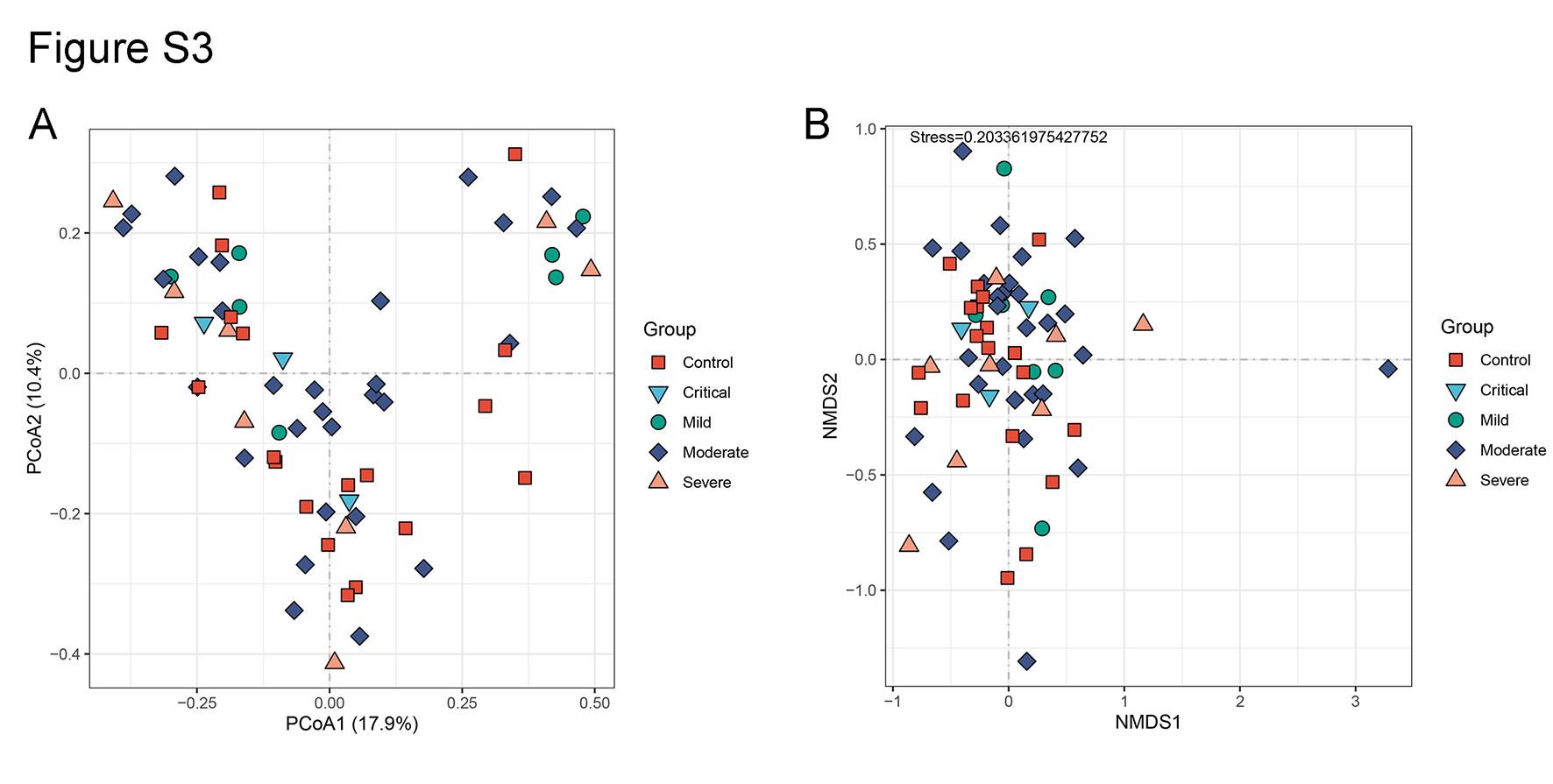

Supplement: Supplementary Figure S3 — The β diversity of various clinical types. [file Image_3.TIF]

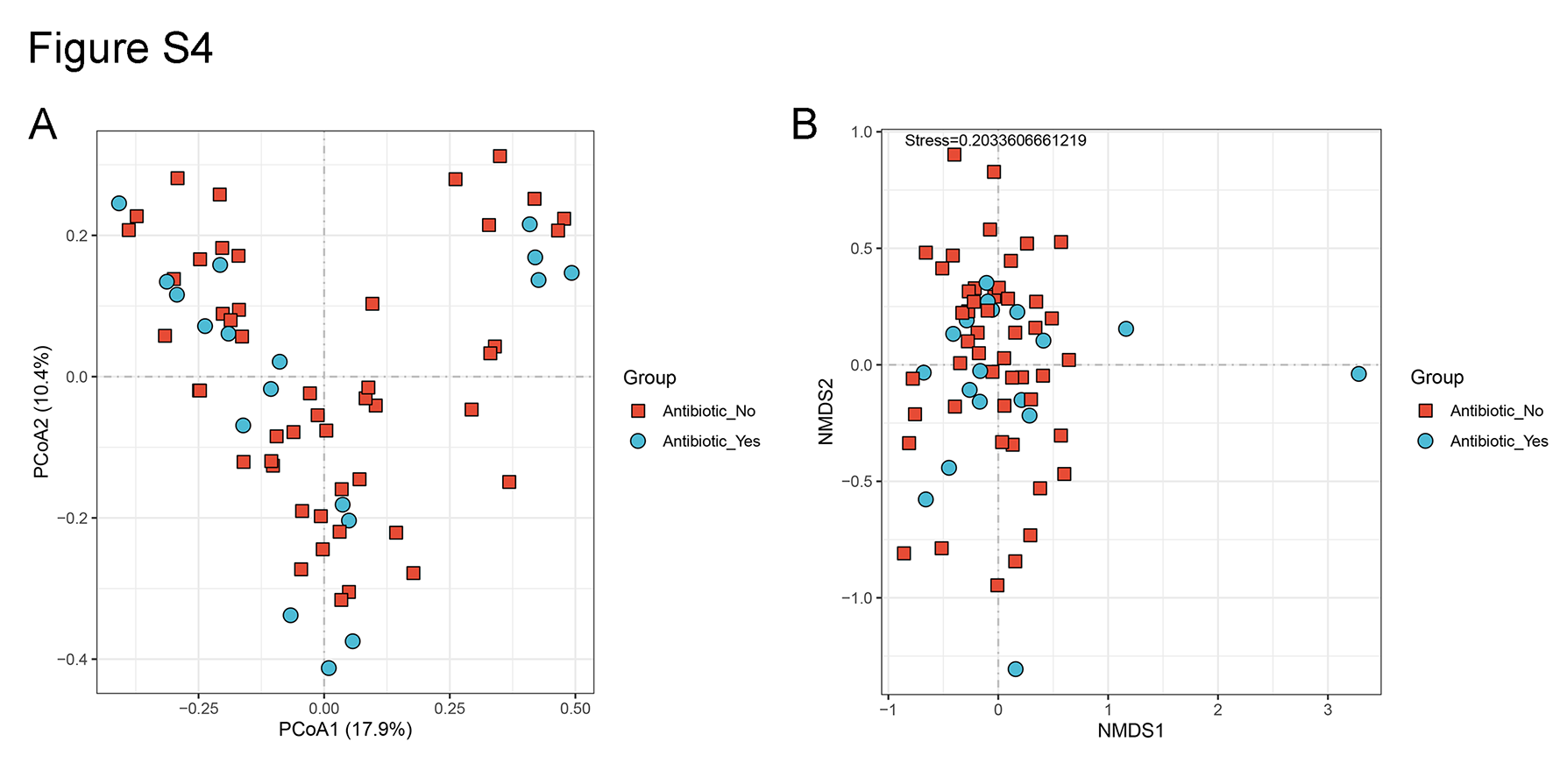

Supplement: Supplementary Figure S4 — The β diversity of ABX-treated and non-treated patients. [file Image_4.TIF]
